# Evolution of Drought–Flood Abrupt Alternation and Its Impacts on Surface Water Quality from 2020 to 2050 in the Luanhe River Basin

**DOI:** 10.3390/ijerph16050691

**Published:** 2019-02-26

**Authors:** Wuxia Bi, Baisha Weng, Zhe Yuan, Yuheng Yang, Ting Xu, Dengming Yan, Jun Ma

**Affiliations:** 1 State Key Laboratory of Simulation and Regulation of Water Cycle in River Basin, China Institute of Water Resources and Hydropower Research, Beijing 100038, China; biwuxia_1992@163.com (W.B.); yuanzhe_0116@126.com (Z.Y.); sduyyh@126.com (Y.Y.); xuting900515@163.com (T.X.); 18519500795@163.com (D.Y.); 18303011501@163.com (J.M.); 2 College of Hydrology and Water Resources, Hohai University, Nanjing 210098, China; 3 Changjiang River Scientific Research Institute, Wuhan 430010, China; 4 College of Environmental Science and Engineering, Donghua University, Shanghai 201620, China; 5 School of Water Conservancy and Hydroelectric Power, Hebei University of Engineering, Handan 056021, China

**Keywords:** drought–flood abrupt alternation, tempo-spatial evolution, surface water quality, model simulations, Luanhe River Basin

## Abstract

It has become a hot issue to study extreme climate change and its impacts on water quality. In this context, this study explored the evolution characteristics of drought–flood abrupt alternation (DFAA) and its impacts on total nitrogen (TN) and total phosphorous (TP) pollution, from 2020 to 2050, in the Luanhe river basin (LRB), based on the predicted meteorological data of the representative concentration pathways (RCPs) climate scenarios and simulated surface water quality data of the Soil and Water Assessment Tool (SWAT) model. The results show that DFAA occurred more frequently in summer, with an increasing trend from northwest to southeast of the LRB, basically concentrated in the downstream plain area, and the irrigation area. Meanwhile, most of the DFAA events were in light level. The incidence of TN pollution was much larger than the incidence of TP pollution and simultaneous occurrence of TN and TP pollution. The TN pollution was more serious than TP pollution in the basin. When DFAA occurred, TN pollution almost occurred simultaneously. Also, when TP pollution occurred, the TN pollution occurred simultaneously. These results could provide some references for the effects and adaptation-strategies study of extreme climate change and its influence on surface water quality.

## 1. Introduction

The term “drought–flood abrupt alternation (DFAA)” was first proposed in 2006 and refers to persistent drought for certain continuous days in one basin or region, followed by sudden heavy precipitation, resulting in steep river water and farmland waterlogging [1]. The DFAA event is an extreme hydrological event in which drought and flood occur alternately in the short term. Affected by global climate change, the extreme non-uniformity of precipitation on a time scale is more prominent in a basin or region, and the frequency and intensity of DFAA events have also increased significantly, such as in south-west Australia, and in the middle and lower reaches of the Yantze River in China. [2,3]. Therefore, DFAA has become one of the hot issues in relevant fields, such as meteorology, hydrology, and agriculture.

Current research of DFAA, includes mechanism analysis, evolution characteristics and disaster damage of DFAA events. The mechanism analysis shows that DFAA events are closely related to atmospheric circulation, monsoon and subtropical highs, and topographical conditions of the basin [4,5,6]. Hastenrath et al. [7] studied the atmospheric circulation mechanisms of the drought and flood anomalies in the boreal autumn ‘‘short rains’’ season in eastern equatorial Africa from 2005 to 2008. Wu et al. [8] and Feng et al. [9] simulated the drought and flood events in the middle and lower reaches of the Yangtze River in late spring and early summer of 2011, based on the ERA-40 re-analysis data and the NOAA-Hysplit model, indicating that the severe droughts were affected by the Ryanna event and the abnormal cold sea temperature control in the equatorial Indian Ocean. With the weakening of the cold sea temperature in the equatorial central and eastern Pacific and the Indian Ocean, abnormal precipitation occurred, which was the main cause of the drought and flood events. Yang et al. [10] also analyzed the characteristics of droughts and floods in the middle and lower reaches of the Yangtze River in 2011. In addition, some other researchers, such as Shen et al. [11], Li et al. [12], and Ma et al. [13], used statistical methods to explore the correlations between DFAA events and large-scale circulation, sea temperature anomalies, and low-frequency circulations. The central-eastern region or basin of China is prone to recurrence of DFAA, and presents different evolution characteristics [14,15,16]. Yan et al. [17] systematically analyzed the evolution characteristics of drought and flood events, based on the daily precipitation data of 171 meteorological stations in Huang-Huai-Hai River Basin from 1961 to 2011. The results show that, as the drought frequency increases, some areas of the basin become more vulnerable to extreme precipitation processes. Research on disaster damage, caused by droughts and floods, has focused on the study of crop physiological characteristics and regional ecological environment [18,19,20,21]. The former relevant studies mainly focus on the meteorological significance of DFAA, while there is relatively scant research on the characteristics analysis, based on the meteorological–hydrological coupling system and the impacts of DFAA on the ecological environment.

Several researchers have carried out related research work on the effects of single drought or single flood events on the migration and transformation of watershed pollutants, as well as the surface water quality [22,23,24,25,26]. However, a study looking at the impact of DFAA events on surface water quality is still in its infancy [26,27]. The eutrophication of water bodies has become a global concern. Nitrogen and phosphorus are two of the most important factors affecting the eutrophication of surface water bodies [28,29], especially in freshwater bodies [30]. Therefore, it is of great significance to study the DFAA impacts on surface water quality, especially the pollution of nitrogen and phosphorus.

According to the “2016 State of the Environment,” the water pollution in the Haihe River Basin (including the Luanhe River Basin (LRB)) is still serious, although the surface water quality has improved compared to previous years [31]. According to the Environmental Quality Standards for Surface Water (GB3838-2002), there are about five classes of water quality. The water quality in Class IV is applicable to the general industrial protection areas and recreational areas with indirect contact to humans, while water quality below Class IV does not have much use. The monitoring results showed that, polluted rivers with water quality in Class IV and below accounted for 43.2% of all evaluated rivers in the last ten years [32]. The main pollution indicators of surface water in the LRB were ammonia nitrogen, total nitrogen (TN), and total phosphorus (TP) [33]. As DFAA could lead to sudden water pollution incidents in the LRB, it is of great importance to study the evolution characteristics of DFAA and its impact on surface water quality in the LRB.

**Figure 1:**
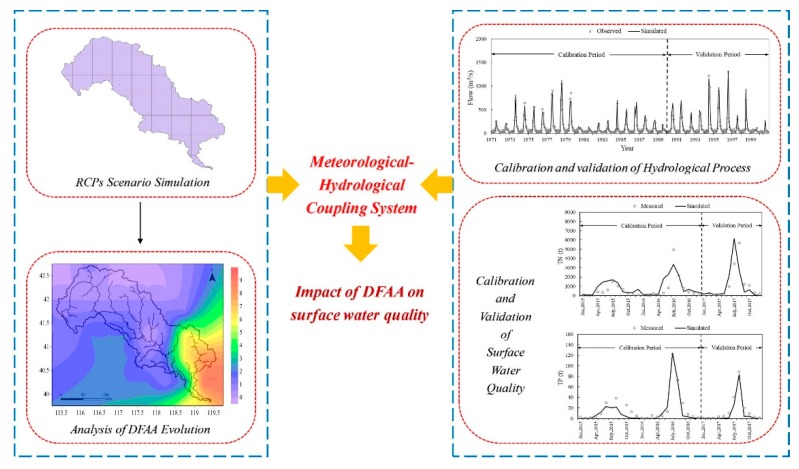
The framework of this study.

The main objectives of our study were to: (i) establish a systematic evaluation standard of DFAA in the LRB; (ii) predict the spatial and temporal evolution characteristics of DFAA from 2020 to 2050 in the LRB; and (iii) investigate the impacts of DFAA events on TN and TP pollution. Under three Representative Concentration Pathways (RCPs) climate scenarios, future climate change was predicted. And the evolution characteristics of total nitrogen (TN) and total phosphorus (TP) load in the LRB were investigated, which were based on the calibration and validation of the hydrological calibration process and water quality of the SWAT model. The research process contains four parts (Figure 1): (i) selection of a future climate scenario based on RCPs; (ii) analysis of the tempo-spatial changes of DFAA in the LRB by a set of criteria proposed; (iii) simulation of TN and TP load with calibration and validation of the SWAT model; and (iv) evaluation of the impacts of DFAA on surface water quality by calculating the incidence of TN and TP pollution. The broad implication of the present research is to better understand the future evolution trends of DFAA in the LRB. The impact analysis of DFAA on surface water quality can guide the adaptation strategies for the changed environment. The evolution mechanism of DFAA and the corresponding surface water quality changes from 2020 to 2050 would provide references for the future planning of disaster preventions, agricultural development, and so on.

## 2. Materials and Methods

### 2.1. Study Site

**Figure 2:**
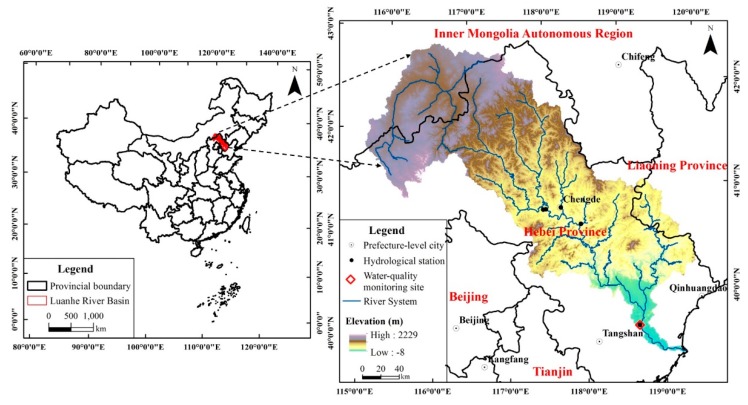
The Luanhe River Basin.

The LRB is located at the northern Haihe River Basin, with geographical scope of 39°10′–42°30′ N, 115°30′–119°15′ E (Figure 2). As one of the four major rivers in the Haihe River Basin, the Luanhe River flows through 27 cities and countries in Hebei Province, Inner Mongolia Autonomous Region and Liaoning Province, with watershed area of approximately 44,750 km^2^. The elevation of the LRB decreases from north to south, with a landform of plateau, mountains and plain, respectively [34,35].

The LRB experiences a humid, semi-humid, and semi-arid temperate continental monsoon climate from southeast to northwest. The region has the characteristics of four distinct seasons, significant monsoon, concentrated precipitation, rain and heat over the same period, and a complex climate. The multi-year average water surface evaporation in the basin is approximately 950 to 1150 mm. The annual precipitation of the LRB is between 390 to 800 mm, with significant heterogeneous seasonal distribution. The precipitation is concentrated in summer, of 260 to 560 mm, accounting for 67% to 76% of the annual precipitation. Thus, DFAA typically occurs at the turn of spring to summer.

### 2.2. RCPs and SWAT Model

Future climate predictions are based on the scenarios considering the emissions of greenhouse gases and aerosols. Representative concentration pathways (RCP) are climate scenarios developed in the Intergovernmental Panel on Climate Change (IPCC) Fifth Assessment Report, including RCP2.6, RCP4.5, RCP6.0, and RCP8.5. According to existing research [36,37], three climate scenarios—RCP2.6, RCP4.5, and RCP8.5—were selected as future climate prediction scenario models to analyze future climate change in the LRB from 2020 to 2050. RCP2.6 is the scenario that limits the average temperature increase lower than 2.0 °C, and the radiative forcing peak appears before 2100 then drops to 2.6 W·m^−2^ in 2100. RCP4.5 is to stabilize the radiative forcing at 4.5 W·m^−2^. RCP8.5 assumes the largest population, a low rate of technological innovation and slow energy improvement, with the highest greenhouse gas emission, the radiative forcing rises to 8.5 W·m^−2^ in 2100. Each climate scenario was based on the interpolated, revised results of five sets of global climate scenarios (GFDL-ESM2M, HADGEM2-ES, IPSL-CM5A-LR, MIROC-ESM-CHEM and NORESM1-M) provided by the Inter-Sectoral Impact Model Inter-comparison Project (ISI-MIP). The evaluation and optimization of climate prediction scenario models have been described in earlier studies [36,37].

ArcSWAT2012 (a public domain model jointly developed by the United States Department of Agriculture (USDA) Agricultural Research Service and Texas A&M AgriLife Research), as a distributed hydrological model, has been widely used to assess the long-term impacts of different climatic conditions and land cover changes on sediment, nutrients and so on. In this study, the LRB was divided into 88 sub-basins, with the smallest catchment area threshold defined as 250 km^2^. The model construction, calibration, and validation are essential and complex. The transformation of different databases [36,38,39,40], the model construction [36], the calibration and validation details of the hydrological process [36,41] and the surface water quality [42] can be found in the corresponding references.

Precipitation and temperature are considered as the main elements of future climate change in this paper. It is assumed that the future point source emissions, withdrawn water, cultivated land areas, and irrigation systems are maintained at the current level.

### 2.3. Data Sources

To explore the evolution characteristics of the DFAA and its impacts on surface water quality in the LRB from 2020 to 2050, this study applied representative concentration pathways (RCPs) for future climate prediction and the SWAT model for surface water quality simulation. The input data mainly contains four types, which are topography, meteorology, hydrology, and water quality. The topographical data include digital elevation models (DEM), land use and soil. The meteorological data include the historical daily data from 1963 to 2017, and future prediction data from 2020 to 2050, based on the simulation results of a future climate prediction scenario model. The hydrological data include the monthly observed flow data from 1970 to 2000 on five hydrological stations. The surface water quality data include monthly measurements of TN and TP, from 2015 to 2017 in the downstream water quality monitoring site, Luanxian Station. The samples were analyzed on the basis of the Environmental Quality Standards for Surface Water (GB3838-2002) [43]. The specific information of the data is shown in Table 1.

### 2.4. Data Analysis

#### 2.4.1. Evaluation of DFAA

The analysis of DFAA is based on the daily precipitation data, which includes three steps: Dividing the drought levels, classifying the flood levels, and determining the DFAA levels.

The number of continuous rainless days is used to identify the drought period and the drought level. The classification criteria are determined by the Standard of Classification for Drought Severity (SL424-2008) (Table 2) [44]. A rainless day refer to the daily precipitation amount less than 3 mm, 5 mm, 3 mm and 3 mm in spring (March to May), summer (June to August), autumn (September to November), and winter (December to February), respectively. If there are rainless days across the season, the drought level is determined by the season with more than half of the rainless days.

The flood levels are determined by the total precipitation amount within 5 days after the droughts. In one precipitation process, when the precipitation amount far exceeds the infiltration amount, it is considered that a flood will occur. The infiltration amount is the product of the precipitation amount and the infiltration coefficient. The main characteristic of the DFAA is the sudden change from drought to flood. Therefore, the DFAA is mainly concerned with the flood caused by the short-term and intensive precipitation processes. When classifying the flood level, the annual maximum *n*-day precipitation is applied to calculate the threshold of infiltration in the DFAA (Equation (1)).

(1) $$I_{n}=\alpha P_{n}$$

where $I_{n}$ is the infiltration amount of continuous precipitation for n days, mm. n is the number of continuous rainy days. α is the infiltration coefficient, mainly affected by precipitation, groundwater depth, underlying surface conditions, and evaporation. Previous studies show the infiltration coefficient is about 0.23 in the North China Plain and the Huang-Huai-Hai Plain, i.e., α equals to 0.23 in this study [45]. $P_{n}$ is the multi-year average of the annual maximum n-day precipitation, mm. To calculate $P_{n}$, we analyzed the historical meteorological data (from 1963 to 2017) of 46 meteorological stations in and around the LRB, and obtained the infiltration amount of continuous *n*-days precipitation ($I_{n}$).

To determine the DFAA levels, the key issue is to classify the flood levels. The flood levels are based on the precipitation threshold, which is in accordance with the infiltration threshold. Equations (2) and (3) calculate the precipitation threshold.

(2) $$PT_{jn}=kI_{n}$$

(3) $$k=k_{0}+j+\beta \left(n-1\right)$$

where $PT_{jn}$ is the precipitation threshold, mm. j is the flood level, when j is equal to 0, 1, and 2, the corresponding flood level is light flood, moderate flood, and severe flood. $k_{0}$ is an undetermined coefficient. Referring to the historical flood data of the LRB, when $k_{0}$ = 3, it is considered that the daily precipitation in the basin reaches the light flood level. k is the corrected value of $k_{0}$, related to the flood level and cumulative rainy days. β is an undetermined coefficient, when j is equal to 0, 1, and 2, β is equal to 0.5, 0.7, and 1, respectively.

The grade of DFAA is judged according to the drought level and the precipitation level after drought. The combination of three drought levels and three flood levels can give nine types of DFAA. The specific types are shown in Table 3.

Based on the types of DFAA, droughts and floods are assigned to the scales 1, 2, and 3 to correspond with the level of light, moderate, and severe, respectively. The DFAA level is judged by the average value of drought level and flood level (Table 3). When the average value is lower than 2, equal to 2, and larger than 2, the DFAA occurs in light level (light drought-light flood, light drought-moderate flood, moderate drought-light flood), moderate level (light drought-severe flood, moderate drought-moderate flood, severe drought-light flood), and severe level (moderate drought-severe flood, severe drought-moderate flood, severe drought-severe flood), respectively. The higher the level, the more serious the DFAA.

#### 2.4.2. Evaluation of DFAA Impacts on Surface Water Quality

The incidence of water pollution was applied to assess the surface water quality situation, which is a single-factor evaluation method. In this study, TN and TP were chosen as the surface water indicators. According to the Environmental Quality Standards for Surface Water (GB3838-2002), the concentration threshold of TN in Class III (water quality is applicable to secondary reserve of centralized drinking water sources, general fish protection areas and swimming areas) and Class IV (water quality is applicable to general industrial protection areas and recreational areas with indirect contact to humans) is 1.0 mg/L, and 1.5 mg/L, respectively; the concentration threshold of TP in Class III and Class IV is 0.2 mg/L, and 0.3 mg/L, respectively. For the unified analysis, the surface water can be considered contaminated with TN and TP concentrations greater than Class IV. Meanwhile, we analyzed the TN and TP pollutions with concentrations greater than Class III. Equation (4) calculates the incidence of water pollution.

(4) $$p=\frac{n_{p}}{N}$$

where p is the incidence of water pollution, $n_{p}$ is the number of polluted months during the study period, N is the total number of months.

To assess the DFAA impacts on TN and TP, the incidence of water pollution was explored in DFAA months, normal months (without DFAA), and total months, respectively. To further study the impacts of different DFAA levels on TN and TP pollution, the incidence of water pollution in DFAA months under different levels was calculated.

## 3. Results

### 3.1. Model Calibration and Validation

**Figure 3:**
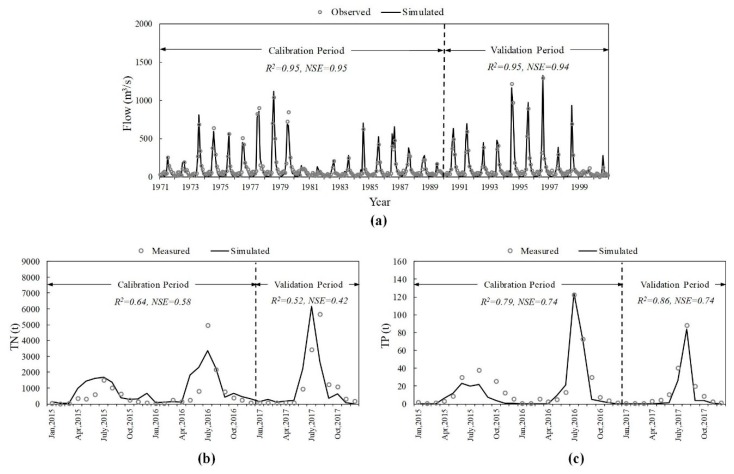
The Soil and Water Assessment Tool (SWAT) model calibration and validation results of (**a**) hydrological process of the Luanxian Station; (**b**) total nitrogen (TN) load; (**c**) total phosphorus (TP) load.

The flow data of five hydrological stations (Figure 2) was applied for the hydrologic calibration and validation process in the SWAT model. As the Luanxian Station is the monitoring station for both hydrology and water quality (Figure 2), the hydrological calibration and validation results of this station are plotted with the article length limit. For the hydrological process, R^2^ and NSE were 0.95 and 0.95 during the calibration period, and 0.95 and 0.94 during the validation period (Figure 3a). The measured water quality data, from 2015 to 2017, were applied for the calibration and validation of TN and TP. Figure 3b,c) show the water quality calibration and validation results. For TN, R^2^ and NSE were 0.64 and 0.58, during the calibration period, and 0.52 and 0.42, during the validation period. For TP, R^2^ and NSE were 0.79 and 0.74, during the calibration period, and 0.86 and 0.74, during the validation period.

### 3.2. Determination of DFAA Levels in the LRB

Table 4 shows the calculation results of the $I_{n}$ of *n*-days precipitation in the LRB. The $I_{n}$ of continuous 1-day, 2-days, 3-days, 4-days, and 5-days precipitation in the LRB is 17 mm, 18 mm, 19 mm, 20 mm, and 22 mm, respectively.

According to Equations (1)–(3), the classification of flood levels in the LRB can be obtained (Table 5). The lower limit of light flood level for continuous 1-day, 2-days, 3-days, 4-days, and 5-days precipitation in the LRB is 50 mm, 60 mm, 75 mm, 90 mm, and 110 mm, respectively. The lower limit of moderate flood level for continuous 1-day, 2-days, 3-days, 4-days, and 5-days precipitation in the LRB is 70 mm, 85 mm, 100 mm, 120 mm, and 150 mm, respectively. The lower limit of severe flood level for continuous 1-day, 2-days, 3-days, 4-days, and 5-days precipitation in the LRB is 85 mm, 110 mm, 130, mm 160 mm, and 200 mm, respectively. When the continuous *n*-days precipitation is larger than the lower limit of the three flood levels, it is considered that the floods occur in distinctive levels.

Combining the Standard of Classification for Drought Severity shown in Table 2, the DFAA levels in the LRB can be determined. In details, the light DFAA refers to the combination of light drought-light flood, light drought-moderate flood, and moderate drought-light flood; the moderate DFAA refers to the combination of light drought-severe flood, moderate drought-moderate flood, and severe drought-light flood; the severe DFAA refers to the combination of mild drought-severe flood, severe drought-mild flood, and severe drought-severe flood.

### 3.3. Evolution Characteristics of DFAA

#### 3.3.1. DFAA in Different Seasons

As the Standard of Classification for Drought Severity varies in different seasons, we conducted the analysis of DFAA in different seasons. Under different RCPs, the frequencies of DFAA were not the same. However, from 2020 to 2050, under all three RCPs selected, there were no DFAA events in winter, most of the DFAA events occurred in summer, and few DFAA events in spring or autumn. There were two DFAA events occurring in spring and autumn under RCP2.6, and one or two DFAA occurring in autumn under RCP4.5, and RCP8.5, respectively. The frequency of DFAA increased from northwest to southeast both in each season and in the whole year. Most of the DFAA was concentrated in the lower reaches of the LRB. Figure 3 plots the specific information about the distribution of DFAA events, from 2020 to 2050, in the LRB.

**Figure 4:**
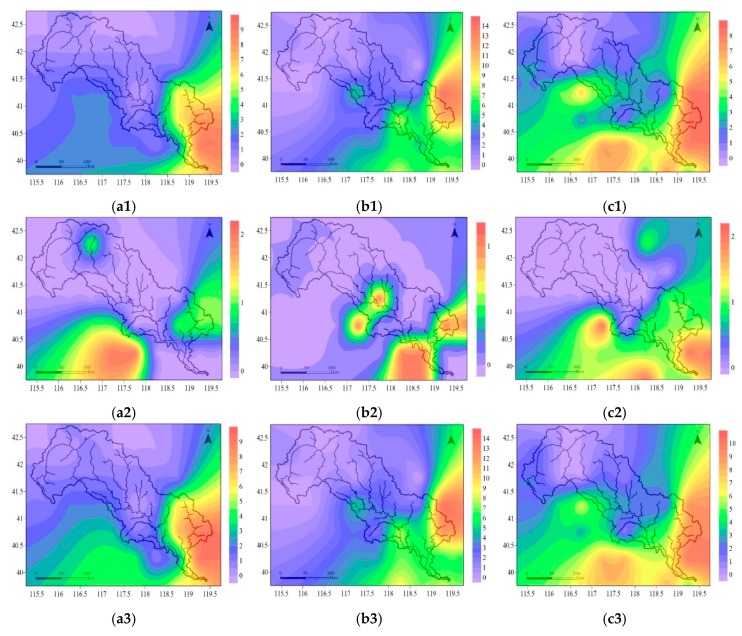
Distribution of drought–flood abrupt alternation events from 2020 to 2050 in the Luanhe River Basin: In summer under RCP2.6 (**a1**), RCP4.5 (**b1**), and RCP8.5 (**c1**); in spring and autumn under RCP2.6 (**a2**), in autumn under RCP4.5 (**b2**) and RCP8.5 (**c2**); in whole year under RCP2.6 (**a3**), RCP4.5 (**b3**), and RCP8.5 (**c3**). RCPs represent the Representative Concentration Pathways. The color bars represent the occurrence frequency of drought–flood abrupt alternation events, for example, the purple means the frequency is zero.

In summer, under RCP2.6, the frequency of DFAA events was zero to two in the upper and middle reaches of the LRB, and varied from three to nine in the downstream (Figure 4(a1)). Under RCP4.5, there were, zero to two, three to five, and six to fourteen DFAA events in the upstream, midstream, and downstream, respectively. The most DFAA occurred in the eastern part of the LRB (Figure 4(b1)). Under RCP 8.5, the frequency of DFAA was zero or one, two to four, five to eight in the upper, middle and lower reaches of the LRB, respectively. The high frequency of DFAA was located in the east and south of the basin (Figure 4(c1)).

In spring and autumn, under RCP2.6, there were almost no DFAA, with one in the northern and eastern part, and one to two in the southern part (Figure 4(a2)). In autumn, under RCP4.5, there was one DFAA in the midstream and downstream (Figure 4(b2)). Under RCP8.5, the frequency of DFAA was zero in the upper and middle reaches of the LRB, while one or two in the downstream (Figure 4(c2)).

On an annual scale, the evolution laws of DFAA were approximately the same as those in summer. Under RCP2.6, there were zero to two DFAA events occurring in the upper and middle reaches of the LRB. The frequency varied from three to nine in the downstream (Figure 4(a3)). Under RCP4.5, the frequency of DFAA was zero to two, three to five, and six to fourteen DFAA events in the upstream, midstream, and downstream, respectively. The most DFAA occurred in the east of the LRB (Figure 4(b3)). Under RCP8.5, there were zero or one, two to four, five to eight DFAA events occurring in the upper, middle and lower reaches of the LRB, respectively (Figure 4(c3)).

#### 3.3.2. DFAA in Different Levels

In this study, the DFAA events in the LRB were classified on three levels. The frequency evolution laws of DFAA had differences under three RCPs. The same as the evolution characteristics in different seasons, the frequency of DFAA increased from northwest to southeast in all three DFAA levels. The DFAA events occurred more frequently in the lower reaches of the LRB. Meanwhile, most of the DFAA events were in light level. Figure 4 shows the distribution of DFAA events in different levels from 2020 to 2050 in the LRB.

**Figure 5:**
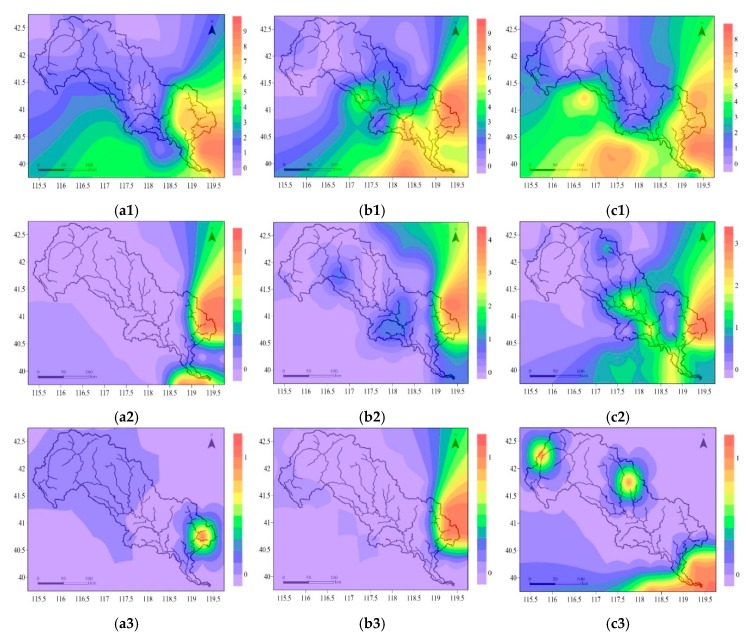
Distribution of drought–flood abrupt alternation events, from 2020 to 2050, in the Luanhe River Basin: In light level under RCP2.6 (**a1**), RCP4.5 (**b1**) and RCP8.5 (**c1**); in moderate level under RCP2.6 (**a2**), RCP4.5 (**b2**), and RCP8.5 (**c2**); in severe level under RCP2.6 (**a3**), RCP4.5 (**b3**), and RCP8.5 (**c3**). RCPs represents the representative concentration pathways. The color bars represent the occurrence frequency of drought–flood abrupt alternation events, for example, the purple means the frequency is zero.

For the light DFAA level, under RCP2.6, the frequency of DFAA events was zero or one in the upstream, one or two in the middle reaches of the LRB, and varied from four to nine in the downstream (Figure 5(a1)). Under RCP4.5, there were zero or one, two to four, and five to nine DFAA events in the upstream, midstream, and downstream, respectively (Figure 5(b1)). Under RCP8.5, the frequency of DFAA was zero or one, two to four, five to eight in the upper, middle and lower reaches of the LRB, respectively. The high frequency of DFAA was located in the eastern and southern part (Figure 5(c1)).

For the moderate DFAA level, under RCP2.6, there was almost no DFAA in the upper or the middle reaches of the LRB, while there were one or two in the downstream, especially in the north and east of the basin (Figure 5(a2)). Under RCP4.5, the frequency of DFAA in the upstream, midstream and downstream was zero or one, zero or one, and one to four, respectively (Figure 5(b2)). Under RCP8.5, there were zero or one, one or two, and zero to three DFAA in the upper, middle, and lower reaches of the LRB, respectively (Figure 5(c2)).

For severe DFAA level, there was almost no occurrence possibility of DFAA events. Under RCP2.6 and RCP4.5, there were almost no DFAA in the upstream and midstream, but one in the eastern downstream (Figure 5(a3),(b3)). Under RCP8.5, maybe one DFAA event could have occurred in the northern part of both the upstream and midstream, also the outlet of the basin in the downstream (Figure 5(c3)).

### 3.4. The Relationship between DFAA and Water Quality

The incidence of water pollution was analyzed to reveal the relationship between DFAA and indicators of water quality. Table 6 plots the incidence of water pollution, based on the Environmental Quality Standards for Surface Water (GB3838-2002) of Class IV under three RCPs. We calculated the incidence of water pollution in the condition of DFAA months, normal months and total months. The incidence of TN pollution, TP pollution, simultaneous occurrence of TN and TP pollution showed similar variation laws in all RCPs, that is, the incidence of pollution increased according to the following order: Normal months < total months < DFAA months. The incidence of TN pollution was much larger than the incidence of TP pollution and the simultaneous occurrence of TN and TP pollution, indicating that TN pollution is more serious than TP pollution in the LRB. For TN pollution, the incidence of pollution was 94.6% to 97.3%, 67.5% to 69.3%, and 70.2% to 72.3% in DFAA months, normal months, and total months, respectively. For TP pollution, in RCP4.5, the incidence of pollution was 2.9%, 0.6%, and 0.8% in DFAA months, normal months, and total months, respectively. The incidence of TP pollution and simultaneous occurrence of TN and TP pollution was the same in DFAA months, normal months and total months, which reveals that when TP pollution occurs, the TN pollution occurs simultaneously. In RCP2.6 and RCP8.5, there was no possibility of TP pollution and simultaneous occurrence of TN and TP pollution.

Table 7 shows the incidence of water pollution with the water quality standard of Class III under three RCPs, the variation laws were the same as the pollution incidence with the standard of Class IV. The incidence of TN pollution, TP pollution, simultaneous occurrence of TN and TP pollution showed similar variation laws in all RCPs, with increasing order: Normal months < total months < DFAA months. The incidence of TN pollution was much larger than the incidence of TP pollution and simultaneous occurrence of TN and TP pollution. The incidence of the TP pollution, and simultaneous occurrence of TN and TP pollution was approximate. For TN pollution, the incidence of pollution was 97.1% to 97.3%, 77.0% to 77.5%, and 78.5% to 79.3% in DFAA months, normal months, and total months, respectively. For TP pollution, the incidence of pollution was 8.1% to 13.5%, 0.9% to 1.5%, and 1.6% to 2.7% in DFAA months, normal months, and total months, respectively. The incidence of TP pollution and simultaneous occurrence of TN and TP pollution was the same in DFAA months, normal months and total months, respectively. We can see that the possibility of TN pollution exceeding Class III in the LRB was much more serious than TP pollution. Meanwhile, when TP pollution occurs, the TN pollution occurs simultaneously.

Table 8 presents the incidence of water pollution with the water quality standard of Class IV under different DFAA levels. The incidence of TN pollution showed similar variation laws in all RCPs, that is, the incidence of pollution increased according to the following order: Light DFAA < moderate DFAA = severe DFAA. While the incidence of TP pollution and simultaneous occurrence of TN and TP pollution was much lower. For TN pollution, the incidence of pollution was 92.96% to 97.1%, 100%, and 100% in light DFAA, moderate DFAA, and severe DFAA, respectively. It can be inferred that when DFAA occurs, the TN pollution almost occurs simultaneously. For TP pollution, in RCP4.5, the incidence of pollution was 3.7%, 0.0%, and 0.0% in light DFAA, moderate DFAA, and severe DFAA, respectively. The incidence of TP pollution, and the simultaneous occurrence of TN and TP pollution, was the same in light DFAA, moderate DFAA, and severe DFAA, which reveals that when TP pollution occurs, the TN pollution occurs simultaneously. In RCP2.6 and RCP8.5, there was no possibility of TP pollution and simultaneous occurrence of TN and TP pollution under different DFAA levels.

It can be discerned that the variation laws of the incidence of water pollution with the water quality standard of Class III under different DFAA levels were similar to the pollution incidence with the standard of Class IV (Table 9). The incidence of TN pollution showed the same variation laws in all RCPs, with increasing order: Light DFAA < moderate DFAA = severe DFAA. The incidence of TN pollution was much larger than the incidence of TP pollution and the simultaneous occurrence of TN and TP pollution. The incidence of TP pollution and simultaneous occurrence of TN and TP pollution were approximate. For TN pollution, the incidence of pollution was 92.9% to 97.1%, 100.0%, and 100.0% in light DFAA, moderate DFAA, and severe DFAA, respectively. For TP pollution, the incidence of pollution varied differently in the three RCPs, with the same variation in RCP2.6 and RCP4.5. In RCP2.6 and RCP4.5, the incidence of TP pollution in light DFAA level was 14.3%, and 14.8%, respectively; while both 0.0% in moderate DFAA level and severe DFAA level. In RCP8.5, the incidence of TP pollution was 0.00%, 16.7%, and 66.7% in light DFAA, moderate DFAA, and severe DFAA, respectively. The variation laws of the incidence of simultaneous occurrence of TN and TP pollution were the same to that of TP pollution. Obviously, the possibility of TN pollution exceeding Class III in the LRB was much more serious than TP pollution. Meanwhile, when TP pollution occurs, TN pollution occurs simultaneously.

## 4. Discussion

### 4.1. Evolution of DFAA

According to the prediction results of RCPs, the annual precipitation presented an increasing trend in the LRB, from 2020 to 2050, with significant alternation of wet and dry [41]. Also, the basin experiences semi-arid, semi-humid and humid temperate continental monsoon climate from northwest to southeast. More than two-thirds of the annual precipitation was concentrated in summer. The analysis results of the evolution characteristics show that the frequency of DFAA increased from northwest to southeast, both in each season and in whole year. And most of the DFAA events were concentrated in summer and located in the lower reaches of the LRB. As the downstream of the LRB is in a plain area, it can be seen that DFAA events occur more easily in the plain area. At the same time, downstream of the LRB is the irrigation area where the non-point source pollution can be brought out more easily. The precipitation amount was small in spring and increased largely in the summer, easily causing the DFAA during this time. As the humidity increases from northwest to southeast, the occurrence probability of abnormal precipitation increases from northwest to southeast as well. The evolution of DFAA is in accordance with the prediction of RCPs and the precipitation distribution in the LRB.

For the DFAA in different levels, the DFAA events decreased according to the following order: Light DFAA > moderate DFAA > severe DFAA, indicating that the light drought-light flood, light drought-moderate flood, and moderate drought-light flood occurred more frequently. In addition, the DFAA events occurred more concentrated in the south and east of the LRB, which is similar to the DFAA in different seasons. The prediction results of RCPs showed that in RCP2.6 and RCP4.5, the precipitation had a significant increase in the downstream plain area; in RCP8.5, large precipitation increases were located in the upper and middle reaches. That is the reason that, in RCP2.6 and RCP4.5, the DFAA events occurred more in the downstream; while in RCP8.5, the DFAA events also occurred frequently in the upper and middle reaches.

### 4.2. Impacts of DFAA on Surface Water Quality

The prediction results of RCPs reveal that the precipitation and temperature will increase from 2020 to 2050, compared with the historical period (from 1963 to 2017) [42]. The increase in temperature and extreme precipitation will aggravate water pollution, which directly or indirectly affect the water quality in the basin [46,47]. The IPCC report also records these phenomena [48]. The analysis of the incidence of TN and TP pollution indicates that the water pollution possibility increased when DFAA occurred, and TN pollution is more serious than TP pollution from 2020 to 2050 in the LRB. This can be explained by the following: Precipitation change will affect the occurrence of droughts and floods. A sudden precipitation increase will aggravate atmospheric deposition and surface erosion, also sudden heavy precipitation will increase the scouring effect as surface runoff becomes more evident. This will cause many surface pollutants, especially the non-point source pollutants to enter water bodies [49], then exerting a subtle influence on the water quality. Nitrogen and phosphorus are the two main elements of non-point source pollution [50]. Meanwhile, flood disasters will also increase the sediment amount in the water bodies, affecting the pollutants transport, further influencing water quality [51]. Also, the temperature increase in the future will affect the chemical reaction rate and microbial degradation ability of aquatic organisms [52]. Simulation experiments show that nitrogen and phosphorus are released easily from sediment to bottom water with temperature increase, and lead to an increase in nitrogen and phosphorus concentrations in surface water. The incidence of TP pollution is also much less than TN pollution in the history, which indicates that the variation trend of the incidence of TN and TP pollution in the future is in accordance with that in the history in the LRB.

## 5. Conclusions

The evolution of DFAA and its impacts on surface water quality in the LRB, from 2020 to 2050, were investigated under RCPs simulations. The major conclusions are as follows:

(1) Under RCP2.6, RCP4.5, and RCP8.5, the DFAA occurred more frequently in summer (from June to August) than in autumn or spring, and there were no DFAA events in winter. The frequency of DFAA increased from northwest to southeast in each season and in whole year. The DFAA events were concentrated in the downstream of the LRB, in plain and irrigation areas.

(2) For different DFAA levels, the DFAA in three RCPs decreased according to the following order: Light DFAA > moderate DFAA > severe DFAA. Most of the DFAA events were in light level. Meanwhile, the DFAA events occurred more frequently in the downstream of the LRB in all three RCPs, while some DFAA occurred in the upper and middle streams of the LRB under RCP8.5.

(3) The incidence of TN and TP pollution was applied to evaluate the impact of DFAA on surface water quality. The incidence of TN pollution was much larger than the incidence of TP pollution, and simultaneous occurrence of TN and TP pollution. When DFAA occurred, TN pollution almost occurred simultaneously. Meanwhile, when TP pollution occurred, the TN pollution occurred simultaneously. The incidence of TN pollution, TP pollution, simultaneous occurrence of TN and TP pollution showed similar variation laws in all RCPs, increasing according to the following order: Normal months < total months < DFAA months. For different DFAA levels, the incidence of TN pollution in all RCPs increased as: Light DFAA < moderate DFAA = severe DFAA.

In general, the DFAA occurred more frequently, from 2020 to 2050, in the downstream, located in the southeast of the LRB. Future climate change, such as increases in temperature and extreme precipitation, will exacerbate water pollution, further augmenting the risk of extreme water quality events in future DFAA. The evolution characteristics of DFAA in this study can provide references for future climate change study, and also can support the extreme weather effects and adaptation-strategy study, thus guiding drought resistance, flood control, and pollution control in the LRB. Further studies can focus on the transport and transformation mechanism of nitrogen and phosphorus in different forms, both theoretical analysis and practical application are needed.

## Figures and Tables

**Table 1:** The database and main sources.

Data Type		Scale	Data Source
Topography	Digital Elevation Model (DEM)	Grid 90 m × 90 m	SRTM data (http://srtm.csi.cgiar.org/index.asp)
	Land use	Dataset of 1985, 2000 and 2014	Resource and Environment Data Cloud Platform of the Chinese Academy of Sciences (http://www.resdc.cn)
	Soil	1:1,000,000	The Second National Land Survey (http://www.soil.csdb.cn)
Meteorology	Daily datasets of basic meteorological elements for China’s national surface meteorological stations (v3.0)	46 stations (from 1963 to 2017)	China Meteorological Data Service Center (http://data.cma.cn)
	RCPs	Grid 1° × 1° (from 2020 to 2050)	IPCC Fifth Assessment Report
Hydrology	Monthly observed flow data	5 stations (from 1970 to 2000)	Hydrological Yearbook
Water quality	Monthly TN and TP load	Luanxian Station (from 2015 to 2017)	Measured data

SRTM represents the Shuttle Radar Topography Mission; RCPs represents the representative concentration pathways; TN refers to total nitrogen; TP refers to total phosphorus.

**Table 2:** Standard of classification for drought severity.

Season	Continuous Rainless Days (d)		
	Light Drought	Moderate Drought	Severe Drought
Spring (Mar. to May)	15–30	31–50	>51
Summer (Jun. to Aug.)	10–20	21–30	>31
Autumn (Sept. to Nov.)	15–30	31–50	>51
Winter (Dec. to Feb.)	20–30	31–60	>61

**Table 3:** The combination types of drought–flood abrupt alternation.

Level	Light Flood (LF) 1	Moderate Flood (MF) 2	Severe Flood (SF) 3
Light drought (LD) 1	LD-LF 1	LD-MF 1.5	LD-SF 2
Moderate drought (MD) 2	MD-LF 1.5	MD-MF 2	MD-SF 2.5
Severe drought (SD) 3	SD-LF 2	SD-MF 2.5	SD-SF 3

**Table 4:** The infiltration amount of continuous *n*-days precipitation in the Luanhe River Basin.

n (d)	1	2	3	4	5
$I_{n}$ (mm)	17	18	19	20	22

n is the number of continuous rainy days; $I_{n}$ is the infiltration amount of continuous *n*-days precipitation.

**Table 5:** The classification of flood levels in the Luanhe River Basin.

n (d)	1	2	3	4	5
Light flood	50	60	75	90	110
Moderate flood	70	85	100	120	150
Severe flood	85	110	130	160	200

*n* is the number of continuous rainy days.

**Table 6:** The incidence of water pollution (%) in Class IV under different months.

Scenario	TN Pollution			TP Pollution			TN and TP Pollution		
	DFAA Months	Normal Months	Total Months	DFAA Months	Normal Months	Total Months	DFAA Months	Normal Months	Total Months
RCP2.6	97.30%	69.25%	72.04%	0.00%	0.00%	0.00%	0.00%	0.00%	0.00%
RCP4.5	97.14%	69.73%	72.31%	2.86%	0.59%	0.81%	2.86%	0.59%	0.81%
RCP8.5	94.59%	67.46%	70.16%	0.00%	0.00%	0.00%	0.00%	0.00%	0.00%

**Table 7:** The incidence of water pollution (%) in Class III under different months.

Scenario	TN Pollution			TP Pollution			TN and TP Pollution		
	DFAA Months	Normal Months	Total Months	DFAA Months	Normal Months	Total Months	DFAA Months	Normal Months	Total Months
RCP2.6	97.30%	76.42%	78.49%	13.51%	1.49%	2.69%	13.51%	1.49%	2.69%
RCP4.5	97.14%	77.45%	79.30%	11.43%	1.48%	2.42%	11.43%	1.48%	2.42%
RCP8.5	97.30%	77.01%	79.03%	8.11%	0.90%	1.61%	8.11%	0.90%	1.61%

**Table 8:** The incidence of water pollution (%) in Class IV under different DFAA levels.

Scenario	TN Pollution			TP Pollution			TN and TP Pollution		
	Light DFAA	Moderate DFAA	Severe DFAA	Light DFAA	Moderate DFAA	Severe DFAA	Light DFAA	Moderate DFAA	Severe DFAA
RCP2.6	97.14%	100.00%	100.00%	0.00%	0.00%	0.00%	0.00%	0.00%	0.00%
RCP4.5	96.30%	100.00%	100.00%	3.70%	0.00%	0.00%	3.70%	0.00%	0.00%
RCP8.5	92.86%	100.00%	100.00%	0.00%	0.00%	0.00%	0.00%	0.00%	0.00%

**Table 9:** The incidence of water pollution (%) in Class III under different DFAA levels.

Scenario	TN Pollution			TP Pollution			TN and TP Pollution		
	Light DFAA	Moderate DFAA	Severe DFAA	Light DFAA	Moderate DFAA	Severe DFAA	Light DFAA	Moderate DFAA	Severe DFAA
RCP2.6	97.14%	100.00%	100.00%	14.29%	0.00%	0.00%	14.29%	0.00%	0.00%
RCP4.5	96.30%	100.00%	100.00%	14.81%	0.00%	0.00%	14.81%	0.00%	0.00%
RCP8.5	92.86%	100.00%	100.00%	0.00%	16.67%	66.67%	0.00%	16.67%	66.67%
